# The DOCA-Salt Hypertensive Rat as a Model of Cardiovascular Oxidative and Inflammatory Stress

**DOI:** 10.2174/157340310793566109

**Published:** 2010-11

**Authors:** Abishek Iyer, Vincent Chan, Lindsay Brown

**Affiliations:** 1 School of Biomedical Sciences, The University of Queensland, Brisbane, QLD 4072, Australia; 2 Department of Biological and Physical Sciences, University of Southern Queensland, Toowoomba, QLD 4350, Australia

**Keywords:** Oxidative stress, Inflammation, DOCA-salt, cardiovascular remodelling, fibrosis, hypertrophy.

## Abstract

Oxidative stress and inflammation are two sides of the same coin that are intricately combined to elicit a chronic pathophysiological stress state, especially as seen in cardiovascular remodelling. In this review, we argue that administration of deoxycorticosterone acetate (DOCA) and sodium chloride to uninephrectomised rats, defined as DOCA-salt hypertensive rats, provides a reliable animal model of oxidative and inflammatory stress in the cardiovascular system. The supporting evidence includes pathophysiological and biochemical changes together with pharmacological responses to synthetic and natural compounds that lower the concentrations of reactive free radical species and that curtail inflammatory responses in the cardiovascular system.

## INTRODUCTION

Cardiovascular remodelling is the outcome of chronic pathophysiological stress in the cardiovascular system [1]. The changes include hypertension, hypertrophy and fibrosis, ultimately leading to an enlarged and more rigid myocardium, together with electrical conduction changes in the heart and smooth muscle and endothelial dysfunction in the vasculature [1, 2]. The aetiology of cardiovascular structural remodelling includes complex biochemical pathways associated with an inflammatory reaction and the generation of reactive free radicals [3]. Oxidative stress and inflammation are two sides of the same coin that are intricately combined to elicit a chronic pathophysiological stress state [3]. Understanding these pathways should allow improved interventions in the clinical management of the consequences of cardiovascular remodelling.

The cellular damage induced by superoxide and other reactive oxygen-containing free radicals is defined as oxidative stress [4]. Superoxide (O_2_^−^), hydroxyl (OH^−^), and peroxynitrite (ONOO^−^) are reactive molecules characterized by the presence of unpaired electrons [4, 5]. Reactive oxygen species such as superoxide are produced in the cells of the body by enzymes including xanthine oxidase, cyclooxygenases, lipoxygenases, myeloperoxidases, cytochrome P450 monooxygenase, uncoupled nitric oxide synthase, heme oxygenases, peroxidases, NADPH oxidases and the enzymes of the mitochondrial electron transport chain [5, 6]. Superoxide can rapidly react with nitric oxide (NO) to form peroxynitrite or convert to hydrogen peroxide to form hydroxyl radicals [6]. Superoxide plays important roles in the functioning of normal cells, including cell signalling pathways and may also recruit and activate immune cells, such as neutrophils as part of the microvascular inflammatory response to pathogens [6, 7]. To maintain physiological concentrations, superoxide is removed by enzymes including superoxide dismutases, glutathione peroxidases, catalase and thioredoxin reductase as well as by reaction with small molecule antioxidants including glutathione, ascorbic acid and other dietary components [4, 5]. Increased superoxide concentrations, either by increased production or decreased removal, result in cellular damage [4].

Inflammation is described as the short-term primary response of the body, crucial for tissue repair, involving many complex signals in distinct cells and organ systems to deal with injuries [8]. This response turns pathological when activated for longer periods. Various bioactive mediators such as cytokines and chemokines orchestrate the inflammatory response in association with inflammatory cells [8]. An increased population of activated tissue inflammatory cells producing reactive free radicals can perpetuate oxidative stress [9]. To fuel this process, inflammatory cells such as leukocytes increase expression and activity of pro-oxidant enzymes including myeloperoxidase and NADPH oxidases thereby aiding in the generation of excess reactive oxygen free radicals [4, 6]. Further, redox regulation of inflammatory signalling occurs at several levels, including direct effects of oxidants, modulation by antioxidants, alterations in the redox equilibrium (for example, thioredoxin and the ratio of reduced:oxidized glutathione) and activation of oxidant- and redox-sensitive transcription cofactors such as NF(B and AP-1 [10].

## OXIDATIVE STRESS, INFLAMMATION AND CARDIOVASCULAR REMODELLING

In cardiovascular remodelling, both oxidative stress mediated by reactive free radical species and inflammation following infiltration of immune-inflammatory cells are strongly implicated in inducing hypertension, hypertrophy, fibrosis, conduction abnormalities and endothelial dysfunction in animal models and humans leading to heart failure [3, 6, 11-13]. Oxidative stress probably promotes inflammation [14] and conversely, inflammation-induced damage promotes oxidative stress [15, 16]. The contributions of oxidative and inflammatory stress towards the pathology and progression of cardiovascular structural remodelling have been difficult to separate.

Rats have been used for scientific research for about 150 years; the commonly used Wistar rat strain was developed in 1906. Rat models of cardiovascular disease, especially hypertension and heart failure [17], have been extensively studied to provide insights into the pathogenesis and progression of cardiovascular disease and to investigate possible therapeutic interventions. This suggests that the interactions between oxidative and inflammatory stress and cardiovascular disease may be unravelled with studies on a suitable rat model that mimics both inflammatory and oxidative stress responses. This review will present the arguments that the uninephrectomised Wistar rat treated with deoxycorticosterone acetate (DOCA) and sodium chloride, referred to as DOCA-salt hypertensive rats, reliably models the relationship between oxidative stress, inflammation and cardiovascular disease. 

## DOCA-SALT HYPERTENSIVE RAT MODEL

The administration of a synthetic mineralocorticoid derivative, DOCA, in combination with salt loading in the diet to young adult Wistar rats following surgical removal of one kidney induces hypertension with cardiovascular remodelling characteristic of human volume-overload induced hypertension, especially hypertrophy, fibrosis, conduction abnormalities and endothelial dysfunction [18-24]. Similar cardiovascular remodelling occurs in patients with hypertension and heart failure [3] but these patients are usually not young, nor on a high salt diet, nor taking salt-retaining compounds nor functioning with a single kidney. Many different experimental protocols have been reported to induce DOCA-salt hypertension in the literature including subcutaneous implantation of DOCA pellets [25, 26]. In our studies, 8-9 week old male Wistar rats weighing around 300-330g are anaesthetised with an intraperitoneal injection of Zoletil (tiletamine (25 mg/kg) and zolazepam (25 mg/kg)) together with xylazine (10 mg/kg Rompun) for uninephrectomy; a lateral abdominal incision is made to provide access to the kidney, and the left renal vessels and ureter are ligated. The left kidney is removed and weighed, and the incision site is sutured with sterile suture needles. The sutured site is also clipped with wound healing clips as a precautionary measure as well as to aid faster healing of the incision site. After uninephrectomy, rats are randomized into two groups: uninephrectomy with no further treatment, and uninephrectomy given 1% NaCl in the drinking water with subcutaneous injections of DOCA (25 mg in 0.4 mL of dimethylformamide every fourth day, DOCA-salt rats). Experiments are generally performed 28 days after surgery [27-37].

DOCA-salt rats mimic most of the changes seen in chronic cardiovascular remodelling in humans including hypertension, hypertrophy, fibrosis, electrical conduction abnormalities and vascular hypertrophy and dysfunction. Cardiac hypertrophy is pronounced in the DOCA-salt hearts and seen in both the left and right ventricles. Echocardiographic studies have shown a thickening of the left ventricular posterior wall without any change in the left ventricular chamber diameter suggesting concentric hypertrophy [36]. Cardiac fibrosis and scar tissue formation develops in both the left and right ventricles with increased expression of collagen I and III mRNA [21, 24, 38-41] leading to excessive perivascular and interstitial collagen deposition [21, 24]. The increased scar tissue formation is accompanied by a severe inflammatory insult seen as an increased extravasation of leukocytes into the ventricular tissue. These changes are accompanied by electrical remodelling as an increase in action potential duration at 20%, 50% and 90% of repolarisation [36]. Functional changes include a decrease in E/A flow ratio, cardiac output, contractile and relaxation measurements (+dP/dT, -dP/dT) and an increase in diastolic stiffness in the DOCA-salt hearts [36]. Vascular hypertrophy can be severe in small and large arteries from DOCA-salt hypertensive rats, with a prominent thickening of the media [42]. Smooth muscle and endothelial dysfunction is seen as decreased responses to sodium nitroprusside and acetylcholine respectively, in isolated blood vessels [43-45]. 

## MECHANISMS OF CARDIOVASCULAR REMODELLING IN DOCA-SALT HYPERTENSION

Clinical studies have shown primary aldosteronism or a decrease in renin to aldosterone ratio to be a significant cause of hypertension [16, 46, 47]. The DOCA-salt hypertensive rat model shows a markedly depressed renin-angiotensin system and thus has been regarded as an angiotensin-independent model with decreased circulating plasma renin concentrations [47]. Increased concentrations of aldosterone lead to increased reabsorption of sodium ions and water from epithelial cells in the distal nephron of the kidney, thereby influencing blood pressure levels [47]. Aldosterone binds to the mineralocorticoid receptor, a member of the nuclear receptor family of ligand-dependent transcription factors, thereby also regulating gene transcription. This mineralocorticoid receptor is expressed in other sites, such as vascular smooth muscle cells, cardiac fibroblasts and the brain, thus modifying the classic view that aldosterone acts exclusively on transport epithelia [47]. Increased aldosterone concentrations may activate oxidative stress through an upregulated NADPH oxidase in the DOCA-salt model [48]. The NADPH oxidases (NOX) are a family of 7 members with distinct distributions, consisting of a catalytic subunit, a p22phox subunit, regulatory subunits, activator proteins and a small G-protein. NOX 2, 4 and 5 are found in endothelial cells with NOX 1 and 4 in vascular smooth muscle cells and NOX 2 and 4 in adventitial fibroblasts [49]. Aldosterone induces superoxide generation *via* mineralocorticoid receptor-mediated activation of NADPH oxidase and Rac1 in endothelial cells, thereby contributing to the development of aldosterone-induced vascular injury [48]. NADPH oxidase amplifies the reactive oxygen species formation in the myocardium as its activity increases during heart failure which in turn induces NO synthase uncoupling and xanthine oxidase activity [4]. NADPH oxidase is the major contributor to reactive oxygen species generation in various cardiovascular disease models and its effect is directly related to the increased protein concentrations. The expression of NADPH oxidase and its protein subunits such as Rac1 and p67*phox *were increased during the progression of cardiovascular diseases and heart failure [50]*. *NADPH oxidase activation releasing reactive oxygen species contributed to vascular endothelial dysfunction, apoptosis and inflammation [50]. NADPH oxidase-induced superoxides in sympathetic ganglia were also responsible for increased neurogenic vasoconstriction [51]. Over-expression of p47phox and gp91phox and reduced expression of intracellular superoxide dismutase have also been reported with increased salt loading [52]. These findings suggest that NADPH oxidase is increased and is responsible for increased superoxide production and possibly contributes to the increased blood pressure in the DOCA-salt hypertensive rat [53]. Thus, the administration of the synthetic mineralocorticoid, DOCA, in combination with a high salt intake and uninephrectomy mimics the responses of hyperaldosteronism-induced hypertension.

Although elevated blood pressure may probably be a major effector of cardiac hypertrophy in the DOCA-salt hypertensive rats, neurohumoral factors such as endothelin, vasopressin and sympathetic nerves may play an important independent role in regulating cardiovascular remodelling in these rats [54]. In addition, endothelin-1 concentrations were elevated in the DOCA-salt rat, which also increased NADPH oxidase-induced superoxide production, also contributing to the endothelin-1-induced vasoconstriction [55]. The endothelin system plays an important role in the pathogenesis of DOCA-salt hypertension and associated remodelling [39, 40, 56, 57]. Endothelin-1 gene and prepro-endothelin-1 mRNA and immunoreactive endothelin-1 concentrations in mesenteric resistance arteries and the aorta were increased in DOCA-salt rats [58, 59]. Further evidence for a role of endothelin in the DOCA-salt model is provided by administration of an endothelin antagonist, which not only lowered blood pressure, but also induced reversal of hypertrophic arterial remodelling [60], left ventricular fibrosis and inflammation [39, 40] and renal and cardiac hypertrophy [40]. 

Chronic stimulation of vasopressin V2 receptor, probably by excessive Na^+^ retention, increased basal blood pressure and worsened the development of DOCA-salt hypertension, organ damage and mortality [61]. Collagen III was elevated from day 2 after DOCA induction compared to blood pressure elevation only after day 4, suggesting damage by neurohumoral factors occurs earlier in this model [41]. Catecholamine storage and metabolism were the topics for early studies with DOCA-salt rats [20,22]. Sympathetic neuroeffector transmission, specifically α_2_-adrenoceptors, was impaired in mesenteric arteries of DOCA-salt rats, where noradrenaline is the predominant vasoconstrictor [62]. Endothelial NO synthase (eNOS) expression and activity were down-regulated while ACE and AT1 receptor expression were up-regulated in the left ventricle of DOCA-salt rats [63] suggesting that the local renin-angiotensin and NO systems may be unfavourably modulated in this model of hypertension. Taken together, these findings suggest that, apart from haemodynamic factors, humoral factors also contribute to the cardiovascular remodelling observed in DOCA-salt rats. 

Activation of the mineralocorticoid receptor is also associated with an increased central sympathetic drive with increased release of vasopressin in rats [47]. Schenk & McNeill [64] argue that the sodium retention in DOCA-salt rats alters central neurohormonal pressor baroreflexes, including increased sympathetic nerve activity, baroreflex attenuation and the activation of the brain renin-angiotensin system. Sodium channels or exchangers may be involved in the central responses to DOCA-salt treatment since amiloride and its analogue, benzamil, attenuated hypertension when given centrally but not peripherally [65]. 

## PHARMACOLOGICAL EVIDENCE THAT DOCA-SALT RATS ARE A MODEL OF OXIDATIVE AND INFLAMMATORY STRESS

Another approach to determining whether the DOCA-salt hypertensive rat is an appropriate model for oxidative stress in the cardiovascular system is to determine organ responses to compounds that alter reactive oxygen species concentrations, especially superoxide. If different compounds either decrease superoxide concentrations or increase removal of superoxide by independent mechanisms, but all reduce cardiovascular symptoms, then this strongly suggests that control of superoxide concentrations is the mechanism for the improvement of cardiovascular function. This hypothesis has been tested using the compounds and mechanisms outlined in Fig. (**1)**. 

Using this hypothesis, inhibition of enzyme systems that produce superoxide should improve cardiovascular structure and function in DOCA-salt rats. Small molecules that inhibit NADPH oxidase [49, 66] should also decrease cardiovascular responses in DOCA-salt hypertensive rats. Apocynin (acetovanillone) inhibits the assembly of NADPH oxidase and therefore enzyme activity; it decreased superoxide production in aortic rings from DOCA-salt rats while chronic administration for 28 days decreased systolic blood pressure [67]. In DOCA-salt rats, the sesame lignan, sesamin, decreased NADPH oxidase subunit expression, prevented the increased NADPH oxidase activity, decreased aortic superoxide production and lowered blood pressure [68]. Both NADPH oxidase and xanthine oxidase contributed to vascular superoxide production in DOCA-salt rats; both apocynin and allopurinol as selective inhibitors lowered blood pressure [69]. 

Since superoxide production by NADPH oxidase is increased by angiotensin II and endothelin by activating selective receptors, antagonism of these receptors should then prevent or reverse oxidative damage to the cardiovascular system in DOCA-salt rats. Treatment with captopril (ACE inhibitor) or candesartan (AT1 receptor antagonist) decreased collagen I mRNA and both perivascular and interstitial collagen deposition in the left ventricles as well as the increased stiffness of the ventricle without changing systolic blood pressure [24]. The ET_A_-selective antagonist, A-127722, reversed and prevented cardiac and vascular remodelling in the DOCA-salt rat [35]. Responses included attenuation of the increased blood pressure and ventricular hypertrophy, prevention of monocyte/macrophage accumulation in the left ventricle, attenuation of the increased left ventricular collagen deposition, reversal of the increased ventricular stiffness, decrease of the action potential duration prolongation and improved vascular function [35]. 

Rapid removal of superoxide should also improve the cardiovascular system in DOCA-salt rats if oxidative stress is important in this model. L-arginine is the biological precursor of NO; NO removes superoxide by a very rapid reaction to form the reactive peroxynitrite radical, ONOO^.^. Chronic administration of L-arginine to DOCA-salt rats markedly decreased the structural changes in the heart and improved both cardiac and vascular function suggesting that rapid removal of superoxide is beneficial [34]. Increased ONOO^. ^can nitrate tyrosine resides in two collagen fibres to increase collagen cross-linking and affect cardiovascular function [70]. Aminoguanidine, an inhibitor of collagen crosslinking, prevented these changes in cardiovascular structure and function in the DOCA-salt rats [32]. Superoxide dismutase is the physiological regulator of superoxide removal; acute administration of superoxide dismutase did not change blood pressure in DOCA-salt rats [71]. There are no long-term studies with increased superoxide dismutase to investigate changes in cardiovascular structure or function in DOCA-salt rats. Acute treatment with the superoxide dismutase-mimetic, tempol, did not lower vascular superoxide concentrations but lowered blood pressure by direct inhibition of sympathetic nerve activity [71]. 

Activation of enzymes that remove superoxide or its product, hydrogen peroxide, should also decrease cardiovascular damage in DOCA-salt rats. Selenium is an essential trace element as part of selenoproteins such as glutathione peroxidase and thioredoxin reductase that are important in removing hydrogen peroxide produced by superoxide dismutase [72]. Thus, an increased dietary selenium intake may offer cardioprotection against oxidative stress, for example in adriamycin-induced damage [73]. In DOCA-salt rats, selenium supplementation reduced collagen deposition and passive diastolic stiffness of the left ventricle, suggesting that this intervention indirectly reduces superoxide concentrations in the heart (Adams and Brown, unpublished results). 

Naturally occurring compounds with antioxidant actions *in vivo *acting *via* different mechanisms should all improve cardiovascular function in DOCA-salt rats if oxidative species are the key mediators of damage. Resveratrol is an effective antioxidant *in vivo *by increasing NO synthesis and also maintaining the reduced intracellular redox state *via *the thioredoxin system [74]. Studies on animal models of human disease suggest that resveratrol has the potential to decrease cardiovascular symptoms in patients with myocardial infarction, arrhythmias, hypertension, cardiomyopathies, fibrosis, atherosclerosis, thrombosis and diabetes [74]. In DOCA-salt rats, resveratrol decreased blood pressure, improved cardiac structure and function and improved endothelial-dependent responses to acetylcholine in isolated blood vessels (Chan, Iyer and Brown, unpublished results). Resveratrol may also induce heme oxygenase-1; induction of heme oxygenase-1 with hemin lowered blood pressure, reduced markers of oxidative stress and inflammation and improved renal structure and function in DOCA-salt rats [75]. 

Many other naturally occurring antioxidants are known, including vitamin E (tocopherol), vitamin C (ascorbic acid) and α-lipoic acid. The increased blood pressure and renal damage was ameliorated by treatment of DOCA-salt rats with either vitamin E or C [76]. Treatment of DOCA-salt rats with α-lipoic acid may suppress renal and vascular endothelin overproduction, leading to decreased blood pressure and both renal and vascular protection [77]. In the heart, L-carnitine attenuated remodelling and improved function in DOCA-salt rats, possibly by decreasing the production of excess reactive oxygen species by the mitochondrial respiratory chain [28].

Similar arguments using compounds that inhibit inflammation at different targets can be used to show that the DOCA-salt rat is a model of inflammation-induced damage in the cardiovascular system. This will particularly apply to the development of fibrosis since this process is initiated by infiltration of inflammatory cells into the myocardium. Inhibition of infiltration, for example with fasudil [78] or tranilast [79], attenuated cardiac fibrosis in DOCA-salt rats. Activation of inflammatory cells within the myocardium will produce inflammatory mediators such as the cyclo-oxygenase products of arachidonic acid; aspirin prevented angiotensin II-induced hypertension and oxidative stress [80]. Similar experiments with cyclo-oxygenase inhibitors in DOCA-salt rats have not been reported. However, treatment with a fermented wheat germ extract, Avemar, which inhibits cyclo-oxygenase activity, decreased macrophage infiltration resulting in decreased collagen deposition in the ventricular myocardium, reversed an increased stiffness of the left ventricle and improved cardiac function in DOCA-salt hearts [29]. The complement system is a primary mediator of the inflammatory process with complement factor 5a (C5a) showing potent chemotactic activity. Prevention of the infiltration of inflammatory by selective inhibition of this activity [81] is a potential mechanism to decrease fibrosis in the DOCA-salt rat heart. 

Control of the inflammatory process by controlling the degree of acetylation of nuclear histone and non-histone proteins can be achieved by inhibitors of histone (lysine) deacetylases (HDACs or KDACs). Compounds such as the anti-cancer drug, suberoylanilide hydroxamic acid (SAHA), prevent pro-inflammatory cytokine production with therapeutic effects reported in animal models of inflammatory diseases [82]. Treatment of DOCA-salt rats with SAHA attenuated cardiovascular remodelling, especially fibrosis, and improved cardiovascular function [27]. 

The nuclear factor (B (NF(B) pathway is pivotal in the production of inflammatory mediators, so control of this pathway should decrease fibrosis and improve function in DOCA-salt rat hearts. Fenofibrate, an activator of PPAR-α, suppressed inflammatory gene expression associated with NF(B, prevented myocardial fibrosis and improved cardiac function in DOCA-salt rats [83]. Rosiglitazone as a PPAR-γ agonist inhibited the expression of TGFβ and cyclo-oxygenase-2 in the kidney of DOCA-salt rats; this was associated with decreased blood pressure and the prevention of renal injury, especially tubular injury and glomerulosclerosis, in these rats [84]. Renal injury in DOCA-salt rats was decreased by inhibition of the inflammatory cytokine, TNFα, with etanercept [85]. Similar studies on cardiac damage in DOCA-salt rats are yet to be reported. 

Compounds with pleiotropic anti-inflammatory responses should also decrease cardiac fibrosis and improve cardiac function in DOCA-salt rats. The combined ACE and vasopeptidase inhibitor, omapatrilat, attenuated the development of cardiovascular hypertrophy, inflammation, fibrosis, and ventricular action potential prolongation in DOCA-salt rats [36]. The lipid-lowering HMGCoA reductase inhibitor, rosuvastatin, also attenuated the development of cardiovascular hypertrophy, inflammation, fibrosis and ventricular action potential prolongation in DOCA-salt rats [31]. Pirfenidone, an effective compound against lung inflammation, attenuated cardiac fibrosis and decreased cardiac stiffness without lowering blood pressure in DOCA-salt rats [37]. 

## CONCLUSIONS

The DOCA-salt model rapidly induces cardiovascular remodelling as in chronic hypertension in humans. Interventions that reduce the concentrations of reactive free radicals such as superoxide, either by decreasing production or increasing removal, decrease the remodelling in the DOCA-salt heart and vasculature. Compounds that suppress the inflammatory responses also decrease remodelling, especially fibrosis. Thus, this model emphasises the role of both reactive free radicals and inflammation in the development of cardiovascular remodelling. The DOCA-salt rat provides a suitable model to allow the testing of natural and synthetic compounds with anti-oxidant or anti-inflammatory responses for their effects on cardiovascular remodelling. This provides opportunities for the development of new therapeutic agents for chronic cardiovascular disease. 

## Figures and Tables

**Fig. (1):**
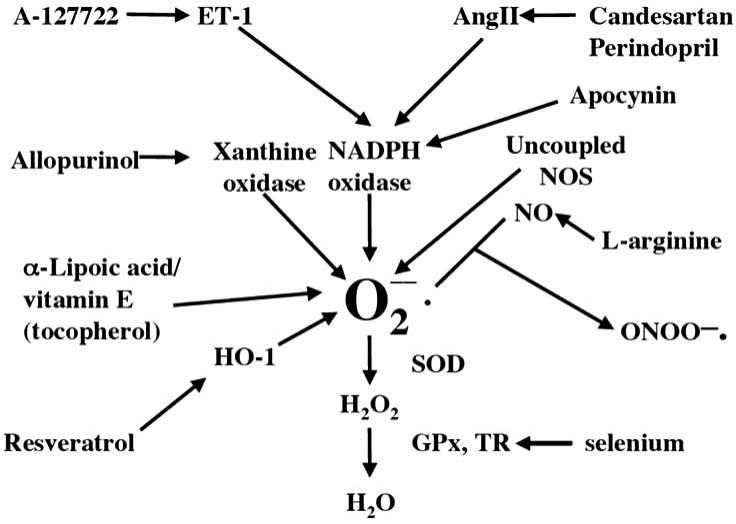
Mechanisms and compounds to decrease formation or increase removal of superoxide. ET-1, endothelin-1; ANGII, angiotensin II; NOS, NO synthase; HO-1, heme oxygenase-1; SOD, superoxide dismutase; GPx, glutathione peroxidase.
